# Patient–physician communication concerning participation in cancer chemotherapy trials

**DOI:** 10.1038/sj.bjc.6601524

**Published:** 2004-01-20

**Authors:** J B Sørensen, P Rossel, S Holm

**Affiliations:** 1 Department Oncology, National University Hospital, 9 Blegdamsvej, DK-2100 Copenhagen, Denmark; 2 Institute of Medical Philosophy and Clinical Theory, University of Copenhagen, Copenhagen, Denmark

**Keywords:** physician–patient communication, satisfaction, chemotherapy, experimental treatment, cancer treatment

## Abstract

Cancer patients demand a high level of involvement in decisions concerning treatment. Many patients are informed about experimental trials, and especially the first consultation may be crucial for the future communication and treatment process. Patients with nonresectable non-small-cell lung cancer or colorectal cancer informed about experimental chemotherapy completed a questionnaire on satisfaction with the communication process, general attitude towards experimental treatments, the substance of information, and personal contact with the physician following their first consultation in a medical oncology unit. Physicians completed a questionnaire on their perception of the patients’ satisfaction. Among 68 physician–cancer patient pairs, 29 patients were informed on chemotherapy in randomised trials and 39 in nonrandomised studies. The general attitude towards experimental treatment was positive or very positive in 71% of patients. Information on the treatment was perceived as completely adequate in 93% of patients informed on randomised and in 67% informed on nonrandomised trials. Physicians underestimated the patients’ satisfaction with the overall communication process, the personal contact, the patients’ perceived sufficiency of the specific treatment information and their ability to decide on study entry. In conclusion, considerable differences were observed between patients informed about experimental chemotherapy in randomised and nonrandomised trials, both with respect to their perception of how adequate the information on the specific treatments were, and whether it was sufficient for decisions on study entry. This study type effect should be accounted for in future evaluations of communication and patient satisfaction. The data also support the fact that cancer patients have a desire for and ability to understand rather detailed and comprehensive treatment information.

Contemporary cancer patients expect a higher level of involvement in the consultation than they have previously, and this preference is now recognised by most physicians (Oken, 1961; Novack *et al*, 1979) This preference for involvement is accompanied by increasing expectations to be fully informed on issues relating to diagnosis, prognosis, treatment options, and their likelihood of success. The physician must also be mindful that the diagnosis of a malignant disease is one of the most significant events in the life of any individual (Siminoff, 1992; Spiro, 1992; Sardell and Trierweiler, 1993). Especially, the first consultation is crucial for the patients' perception of the entire situation and for the future communication and treatment process. Further complications in the communication process may occur when the treatment offered is experimental within a clinical trial. In this case, the patients’ satisfaction with the information process is essential for their understanding of their illness and the complex combinations of treatment and drugs that are used, and also for compliance with instructions and treatment plans (Ley *et al*, 1976; Ley, 1982; Kaplan *et al*, 1996).

Patients are more likely to be satisfied with the physician–patient interaction when physicians provide clear information, are sensitive to the patients’ needs, answer patient questions and do not dominate the exchange (Stiles *et al*, 1979; Smith *et al*, 1981; Blanchard *et al*, 1990; Bertakis *et al*, 1991). Discrepancies between the patients' and the physicians’ expectations of the consultation may possibly influence the outcome and the level of satisfaction.

The aim of this study was to evaluate the physician–patient communication process during the cancer patients' first visit to the medical oncology unit to be informed about the possibility for entry into a clinical study (phase II or Phase III trial) with chemotherapy. The issues under evaluation were: (a) the patients' satisfaction with the communication process as a whole, (b) the physicians’ perception of the patients’ satisfaction with the communication, (c) the influence of the patients’ general attitude towards experimental treatment, and (d) the influence of the physicians’ charge and experience in medical oncology.

## MATERIAL AND METHODS

Eligible patients had nonresectable non-small-cell lung cancer or colorectal cancer and were referred to the medical oncology unit for chemotherapy. Only patients who were informed about treatment within a clinical trial, being either phase II or phase III studies, were included. The clinical studies informed about were accepted by the health authorities and by the ethical committee, while the communication study was accepted by the ethical committee.

The patients were informed about the communication study immediately before their first consultation in the ward. This information was given by a physician other than the physician who should have the consultation with the patient. The information was both verbal and in writing and written informed consent was obtained. The physician to have the consultation also gave written informed consent to participate following verbal and written information.

The attending physician was obliged to perform the following duties during the first consultation: (a) make the patient aware of the malignant diagnosis (all patients were referred from other departments that had diagnosed the malignant disease and that had informed the patient about it), (b) inform about the prognosis with and without therapeutic intervention, (c) inform about the nature, risk, and benefits of the proposed intervention, including mentioning the patients' right to self-determination and hand-out of written information, (d) inform about normal standard treatment outside of the clinical study, and (e) mention the likelihood of success both with the experimental treatment as well as the alternative standard treatment. Informed consent for the clinical treatment study was not to be obtained during the patients' first consultation, as this served solely informative purposes and all patients were informed neither to give nor refuse consent before the following visit.

The attending physician who was to inform the patient during this first consultation could according to the traditional organisation of the hospital wards in Denmark be either a consultant, a resident, or an intern as well. Residents are physicians who have completed a clinical education programme of minimum 5 years together with 8–10 formalised postgraduate courses. Interns are physicians who are in the process of completing this programme and they are in departments of oncology usually already quite skilled, though not yet fulfilling the criteria for being a resident. Very specified and written instructions regarding information of patients are necessary and in routine use, both to cover for the differences in education and for reasons of legislation. Thus, the clinic's usual routine for obtaining consent, a procedure that was similar for standard treatments and for experimental treatments within a clinical trial, was followed: the attending physician provided the patients with both verbal and written information during the patients' first visit to the clinic, and both the patients and their relatives had the opportunity to ask questions. Also, the legislation concerning clinical trials and patients’ autonomy in treatment decisions was described and a written summary of this legislation was given. On the subsequent visit, the patient had a new consultation with the attending physician, during which the patient and the relatives could again ask further questions. The patient was asked to sign the particular written patient information sheet if any treatment were to be embarked on, independent of that being either a standard treatment or an experimental treatment within a clinical trial; one copy of this was kept by the patient and one was kept with the patient record.

With respect to the minimum requirements of knowledge to be conferred to the patients and their relatives concerning the disease, possible complications, treatment possibilities, and prognosis, this was outlined for physicians attending the ward in an eight-page manual concerning colorectal cancer and in an 11-page manual concerning non-small-cell lung cancer, respectively. With respect to possible benefits, side effects, and complications to standard treatments, this was outlined for the physicians in the manual described above and for the patients in the written patient information in addition to the verbal explanation. Similar information concerning experimental treatments in clinical studies was described for the physicians in the experimental protocols and for the patients in the written patient information concerning the study in addition to the verbal explanation. These disease-oriented manuals, protocols on experimental treatments, and written patient information sheets for both standard and experimental treatments facilitated uniform information to patients as far as possible. There was, however, no audit of the information given and some variations in these might still exist. Each consultation during the first visit to the clinic was scheduled for 1 h and the subsequent visit was scheduled for half an hour.

Both the patient and the physician completed a questionnaire of six items and four items, respectively, immediately following the consultation. Each patient–physician pair was unaware of each others’ answers to the questionnaire, as these were collected by another physician. There were no questionnaires for relatives participating in the consultation.

The following patient characteristics were registered: age, gender, diagnosis, accompanied by relatives during consultation, and type of clinical treatment study (randomised or nonrandomised). The patient questionnaire asked about the following six items: satisfaction with the communication process overall, with the personal contact with the physician, with the specific information concerning the experimental treatment, whether the information was sufficient to decide on consent or not to experimental treatment, whether there was sufficient possibility to ask questions, and about the patients’ general attitude to experimental treatment studies not taking the actual one into account. No formal information was sought from the relatives, but the relatives could assist the patient in answering the questionnaire.

The following physician characteristics were recorded: age, gender, charge, duration of experience as physician, and duration of experience in oncology. The physician questionnaire asked about the following four items: the physicians' perception of the patients' satisfaction with the communication process overall, the personal contact with the patient, perception of the patients' satisfaction with the specific information concerning the experimental treatment, and perception on whether the patient may be able to decide on consent or not to experimental treatment. Answers were given according to five-point Likert scales.

## RESULTS

A total of 68 patient–physician pairs were evaluated. These 68 patients’ characteristics are shown in Table 1

**Table 1:** Characteristics of 68 physician–cancer patient pairs

	**Patients, *n*=68**	**Physicians, *n*=25**
	***n*(%)**	
*Gender*		
Male	47 (69%)	14 (56%)
Female	21 (30%)	11 (44%)
		
*Age (years)*		
Median	59	36
Range	37–79	26–48
		
*Diagnosis*		
Lung cancer	37 (54%)	
Colorectal cancer	31 (45%)	
		
*Relatives present*		
No	16 (23%)	
Yes	53 (77%)	
		
*Randomised study*		
No	36 (52%)	
Yes	33 (48%)	
		
*Physicians charge*		
Consultant		3 (12%)
Resident		8 (32%)
Intern		14 (56%)
		
*Experience as physician (years)*		
Median		10
Range		1–18
		
*Experience in oncology (years)*		
Median		4
Range		1–14

. Most patients were men (69%), the median age was 59 years, and the majority had lung cancer (54%). About half of the patients (48%) were informed about treatment within a randomised study, and most patients had followed the advice to be accompanied by a relative (77%). The characteristics of the 25 physicians who participated in the study are shown in Table 1. The median age was 36 years and 56% were males. Three consultants (12%) participated in the study, while the majority of physicians were interns, that is physicians undergoing postgraduate clinical training (56%). The median experience as a physician was 10 years and the median oncologic experience 4 years overall. For interns, these figures were 6 years (range 1–13 years) and 1 year (range 1–4 years), while it was 15 years (range 6–18 years) and 10 years (range 6–14 years) for residents, respectively. All the first consultations evaluated lasted 1 h each.

The patients’ satisfaction with the entire communication during the consultation is shown in Table 2,

**Table 2:** Patients' satisfaction with the entire communication process during thefirst consultation and physicians' perception of their patients' satisfaction

		**Physicians**	
**Evaluation**	**Patients, *n*=68**	**Cons.+Res. (49 pt./phys.pairs)**	**Interns (19 pt./phys.pairs)**
Very good	45 (66%)	13 (27%)	5 (26%)
Good	21 (31%)	21 (43%)	9 (47%)
Acceptable	2 (3%)	14 (29%)	5 (26%)
Poor	0	1(2%)	0
Not acceptable	0	0	0
Unanswered	0		

Abbreviations: Cons.=consultants; Res.=residents; pt./phys. pairs=patient/physicians pairs.

together with the perception by the physicians of how they believed the patients rated the communication. While the patients in 66% of cases rated the communication as ‘very good’ this was only the case for 26% of the physicians’ evaluations. Only 3% of patients’ evaluations rated ‘acceptable’ and none rated ‘poor’. Similar figures for physicians were in contrast 28 and 2%, respectively. When splitting the physicians’ evaluations into a more experienced group (consultants and residents, 49 patient–physician pairs) and a less experienced group (interns, 19 patient–physician pairs), no differences were observed (Table 2).

The personal contact was perceived as ‘very good’ or ‘good’ by 69 and 31% of patients, but was perceived less good by physicians (32 and 50%, respectively). It was even rated as ‘poor’ by the physicians in 4% of cases (Table 3

**Table 3:** Evaluation of the personal contact during first consultation by 68 physician–cancer patient pairs

	**Patients**	**Physicians**
**Personal contact**	***n* (%)**	***n* (%)**
Very good	47 (69%)	22 (32%)
Good	21 (31%)	34 (50%)
Acceptable	0	9 (13%)
Poor	0	3(4%)
Unacceptable	0	0
Unanswered	0	0

). Regarding the satisfaction with the information concerning the experimental chemotherapy within either phase II or phase III trials, most patients (78%) found it ‘completely adequate’. Only 3% found it ‘less acceptable’ and none found it ‘insufficient’ or ‘too detailed’. The corresponding figures for the physicians’ perception of the patients’ evaluation on this subject are shown in Table 4

**Table 4:** Satisfaction of the information on experimental chemotherapy during the first consultation by 68 cancer patients and their physicians' perception of the patients' level of satisfaction

	**Patients**		
**Satisfaction**	**Randomised study (*n*=29)**	**Nonrandomised study (*n*=39)**	**Physicians**
Completely adequate	27 (93%)	26 (67%)	42 (62%)
Acceptable	1 (3%)	8 (21%)	15 (22%)
Less acceptable	0	1 (3%)	1 (2%)
Insufficient	0	0	0
Too detailed	0	0	10 (15%)
Unanswered	1 (3%)	4 (10%)	0

. It is of interest that the physicians in 15% of cases believed that the patients found that it was ‘too detailed’. Table 4 shows the patients’ satisfaction according to whether it was information concerning a randomised phase III study or a nonrandomised phase II treatment. A somewhat higher proportion found the information concerning the randomised study ‘completely adequate’ than was the case for nonrandomised studies, the figures being 93 and 67%, respectively.

The sufficiency of the information with respect to enabling the patients to later decide whether or not to consent on the experimental treatment was evaluated by both patients and physicians (Table 5

**Table 5:** Evaluation of the information on experimental chemotherapy during the first consultation by 68 physician–cancer patient pairs: patients' self-estimation of ability to consent or not and physicians' perception of the patients' ability

	**Patients**		**Physicians**	
**Evaluation**	**Randomised study (29 patients)**	**Nonrandomised (39 patients)**	**Cons.+Res. (49 p/p pairs)**	**Interns (19 p/p pairs)**
Able to decide	29 (100%)	33 (85%)	39 (80%)	15 (79%)
Unable/uncertain	0	2 (5%)	11 (20%)	3 (16%)
Unanswered	0	4 (10%)	0	1 (5%)

Abbreviations: Cons.=consultants; Res.=residents; p/p pairs=physician–cancer patient pairs.

). The evaluation that the information given made the patient ‘able to decide’ was given by 91% of patients, which contrasted somewhat to the physicians’ answers, who in 21% of cases perceived that the patients were ‘unable to decide’. This perceived sufficiency of information was further analysed according to study type (randomised or nonrandomised), and according to physicians’ charge (Table 5). All patients informed about a randomised study felt ‘able to decide’, while the same holds true for 85% of cases with nonrandomised studies. Physicians’ perception on the matter was independent of their charge (Table 5). Even though the majority of patients (52 patients (75%)) indicated that the information given concerning their malignant disease was sufficient, 13 patients (19%) felt that they had not received sufficient information on their illness when the physician started information on treatment options including experimental chemotherapy. Four patients did not respond to this particular question.

The patients’ general attitude towards experimental treatments, that is not the particular study under consideration but their basic feeling about such treatment propositions, is shown in Table 6

**Table 6:** Cancer patients' general attitude towards experimental treatments, divided into 29 patients informed about chemotherapy within a randomised trial and 39 informed about a nonrandomised trial

**General attitude**	**Randomised study (29 patients)**	**Nonrandomised study (39 patients)**
Very positive	7 (24%)	5 (13%)
Positive	15 (52%)	21 (54%)
Neutral	6 (21%)	12 (31%)
Negative	0	0
Very negative	1 (3%)	0
Unanswered	0	1 (3%)

. It is also analysed according to the proposed study type (randomised or nonrandomised) under consideration. Only one patient (2%) was ‘very negative’ about the thought of an experimental treatment, while 66 out of 68 patients (96%) were either ‘neutral’, ‘positive’, or ‘very positive’. No differences were noted among patients informed about randomised or nonrandomised experimental treatments.

## DISCUSSION

Effective patient–physician communication is critical to the informed consent process necessary for appropriate cancer therapy (Siminoff, 1992; Butow *et al*, 1995; Ong *et al*, 1995; Bennett and Alison, 1996), and surveys have shown that patients want a high degree of information and the information usually produces a positive effect (Fallowfield *et al*, 1995; Meredith *et al*, 1996). Others have pointed out that the information needs to be individualised (Butow *et al*, 1997), and also the expectations of the patients have been evaluated in relation to satisfaction with the consultation (Brown *et al*, 1997). A specific, but far from rare, situation is when the responsibility of the physician is the discussion of investigational therapies. Although several standard regimens may have been described in the literature, there is typically a consensus among oncologists that the current state of management is unsatisfactory, and that one reasonable option is to suggest the patient to be enrolled in a prospective clinical trial. In such cases, another dimension in the communication process is added to the already complex situation. This situation was the focus of the current study, which revealed that the attitude of the patients is generally in favour of participation in a clinical trial, with 71% being either positive or very positive, and only 2% being negative (Table 6). This general statement may, however, not necessarily reflect in the patients’ attitude towards a particular experimental treatment being suggested, as this is an area in which the entire actual communication process may influence in addition the patients’ basic attitude towards the subject. The perception of this total communication process was stated as either ‘good’ or ‘very good’ by 97% of patients (Table 2), a figure that might be influenced by factors such as the previously mentioned attitude to experimental treatments in general, or by the actual communication process including the specific information given. Also, the patients’ perception of the personal contact to the physician, which was rated as ‘good’ or ‘very good’ by patients (Table 3), may have contributed to the patients’ impression of the communication process and hence the perception of sufficiency of the information given.

A primary responsibility of the physician is to impart factual, reasonable, and appropriate information so that the patient is appropriately informed and enabled to make reasonable decisions relative to treatment options. Moreover, the data show marked clinical improvement in patients with advanced cancer who are able truly to collaborate with their physicians on account of their thorough understanding of the treatment (Shapiro, 1998). The information on the experimental treatments was perceived as ‘completely adequate’ in 78% of patients, but were accordingly less satisfactory in 22% of cases (Table 4). Especially the latter patient group may be very interesting in order to offer more satisfactory information to future patients. One possible explanation for the latter group is the observation of more patients having the perception of completely adequate information when informed about randomised trials in contrast to those informed on nonrandomised experimental treatment (Table 4). Even though both the verbal and written information on the first subject was more comprehensive than for the apparently more straightforward concept of nonrandomised treatment, this comprehensiveness may be requested by the patients. This concept of comprehensive information is in accordance with a study on 250 patients at an oncology centre in Scotland, which showed that 79% of patients wanted as much information as possible (Meredith *et al*, 1996). The proportion of patients who wanted to know the chance of cure and the side effects of therapy was 91 and 94%, respectively. It is accordingly of interest for future treatment studies that also the current study focusing solely on patients receiving experimental chemotherapy pointed towards more extensive information on the treatment in itself being perceived as beneficial and not burdensome as may otherwise be feared.

An interesting observation in this study was that the physicians underestimated both the patients’ perception of the entire communication process (Table 2), the perceived adequacy of treatment information (Table 4), the patients’ ability to decide on whether or not to participate in the experimental treatment (Table 5), and also perceived a less good contact with the patient than *vice versa* (Table 3). There were no differences between physicians’ perception according to charge (Tables 2 and 5). The reasons for these observations are unclear. One explanation may be that the physicians took a very cautious viewpoint in order not to overestimate their own contribution. Another may be that the patients’ ability to comprehend and the desire to obtain information was truly underestimated. The latter explanation is in accordance with the finding by others suggesting that physicians have difficulties in estimating the amount and type of information that patients want and their effectiveness in imparting information (Blanchard *et al*, 1988; Mackillop *et al*, 1988; Wiggers *et al*, 1990).

Many difficulties are attributed to the evaluation of the communication process. Previous studies have revealed predictors of information and involvement preferences, such as age, gender, and education (Cassileth *et al*, 1990; Hack *et al*, 1994), or situational factors such as purpose and type of consultation (e.g. new patient or later follow-up) and the presence or absence of a companion (Beisecker, 1990; Beisecker and Moore, 1994). In addition, a number of other, hitherto unknown factors may be of influence. This raises a number of methodological deficiencies in the current study, which should be addressed in future research. Firstly, the patients’ answers about their general attitude towards experimental treatment and to other questions related to their expectations for the communication should be given before the consultation, followed by a questionnaire on the perception of the consultation itself. As previously pointed out, these two inherently different types of questions might otherwise influence each other and blur the information obtained. Secondly, the situational factors and the patient-related factors should be either homogeneous or at least registered and accounted for. With respect to situational factors, these were somewhat standardised as all consultations were with new patients having their first visit to the department and all were informed on prefixed subjects, including information concerning experimental chemotherapy to all patients. However, as documented by the current results, differences in the type of investigational treatment (i.e. randomised or nonrandomised trials) under question may influence the patients’ perception of the communication process and should hence either be accounted for or standardised to only one type of experimental treatment under investigation. With respect to patient-related factors, educational status was not recorded, which could be an important predictor of information and involvement preferences, and must as such be accounted for in future studies.

Another difficulty in the evaluation of the communication process is the lack of objective audit concerning both content and form. Thus, the subjective reports of the communication may not necessarily bear much relationship to what objectively happened. This study does not provide an answer to that particular problem, which must be dealt with in subsequent investigations in this field.

In conclusion, the majority of patients (71%) reported a positive or very positive attitude towards experimental chemotherapy, 97% perceived the general communication process during their first visit to the clinic as either good or very good, and all rated the personal contact with the physician as good or very good. Considerable differences were observed between patients informed about randomised and nonrandomised experimental chemotherapy with respect to their perception of how adequate the information on the specific treatment was and whether it was sufficient for decisions on study consent. This should, in future studies, either be accounted for or all patients should be under evaluation for similar treatments. Physicians seem to underestimate the patients’ ability to comprehend and desire to obtain information. These data support reports in the literature pointing towards patients’ desire for and ability to understand rather detailed and comprehensive information.

## References

[bib1] Beisecker AE (1990) Patient power in doctor–patient communication: what do we know? Health Commun 2: 105–122

[bib2] Beisecker AE, Moore WP (1994) Oncologists perceptions of the effects of cancer patients companions on physician–patient interactions. J Psychosoc Oncol 12: 23–39

[bib3] Bennett M, Alison D (1996) Discussing the diagnosis with cancer patients. Postgrad Med J 72: 25–29 8746281 10.1136/pgmj.72.843.25 PMC2398314

[bib4] Bertakis KD, Roter D, Putnam SM (1991) The relationship of physician medical interview style to patient satisfaction. J Fam Pract 32: 175–181 1990046

[bib5] Blanchard CG, Labregue BA, Ruckdeschel JC, Blanchard EB (1990) Physician behaviours, patient perceptions, and patient characteristics as predictors of satisfaction of hospitalised adult cancer patients. Cancer 65: 186–192 2293865 10.1002/1097-0142(19900101)65:1<186::aid-cncr2820650136>3.0.co;2-4

[bib6] Blanchard CG, Labregue MS, Ruckdeschel JC, Blanchard EB (1988) Information and decision making preferences of hospitalised cancer patients. Soc Sci Med 27: 1139–1145 3206248 10.1016/0277-9536(88)90343-7

[bib7] Brown R, Dunn S, Butow P (1997) Meeting patients expectations in the cancer consultation. Ann Oncol 8: 877–882 9358938 10.1023/a:1008213630112

[bib8] Butow PN, Dunn SM, Tattersall MHN (1995) Communication with cancer patients: does it matter? J Palliat Care 11: 34–38 8648521

[bib9] Butow PN, Maclean M, Dunn SM, Tattersall MHN, Boyer MJ (1997) The dynamics of change: cancer patients preferences for information, involvement and support. Ann Oncol 8: 857–863 9358935 10.1023/a:1008284006045

[bib10] Cassileth BR, Zupkis RV, Sutton-Smith K, March V (1990) Information and participation preferences among cancer patients. Ann Intern Med 92: 832–836 10.7326/0003-4819-92-6-832 7387025

[bib11] Fallowfield L, Ford S, Lewis S (1995) No news is not good news: information preferences of patients with cancer. Psychooncology 4: 197–202 11655006 10.1002/pon.2960040305

[bib12] Hack TF, Degner LF, Dyck DG (1994) Relationship between preferences for decisional control and illness information among women with breast cancer: a quantitative and qualitative analysis. Soc Sci Med 39: 279–289 8066506 10.1016/0277-9536(94)90336-0

[bib13] Kaplan SH, Greenfield S, Gandek B (1996) Characteristics of physicians with participatory decision-making styles. Ann Intern Med 124: 497–504 8602709 10.7326/0003-4819-124-5-199603010-00007

[bib14] Ley P (1982) Satisfaction, compliance and communication. Br J Clin Psychol 21: 241–254 7171877 10.1111/j.2044-8260.1982.tb00562.x

[bib15] Ley P, Bradshaw PW, Kincey JA, Atherton ST (1976) Increasing patients satisfaction with communication. Br J Soc Clin Psychol 15: 403–413 1000148 10.1111/j.2044-8260.1976.tb00052.x

[bib16] Mackillop WJ, Steward WE, Ginsberg AD, Steward SS (1988) Cancer patients perception of their disease and its treatment. Br J Cancer 58: 355–3582460120

[bib17] Meredith C, Symonds P, Webster L (1996) Information needs of cancer patients in west Scotland: cross sectional survey of patients views. Br Med J 313: 724–726 8819442 10.1136/bmj.313.7059.724 PMC2352093

[bib18] Novack DH, Freireich EJ, Vaisrub S (1979) Changes in physicians attitudes toward telling the cancer patient. JAMA 241: 890–897 762865

[bib19] Oken D 1961 What to tell cancer patients: a study of medical attitudes. JAMA 175: 1120–1128 13730593 10.1001/jama.1961.03040130004002

[bib20] Ong LM, De Haes JCJM, Hoos AM (1995) Doctor–patient communication: a review of the literature. Soc Sci Med 40: 903–918 7792630 10.1016/0277-9536(94)00155-m

[bib21] Sardell AN, Trierweiler SJ (1993) Disclosing the cancer diagnosis. Cancer 72: 3355–3365 8242563 10.1002/1097-0142(19931201)72:11<3355::aid-cncr2820721135>3.0.co;2-d

[bib22] Shapiro RS (1998) Informed consent. In Principles and Practice of Supportive Oncology, Berger A, Portenoy RK, Weissman DE (eds), pp 785–791. Philadelphia: Lippincott-Raven Publishers

[bib23] Siminoff LA (1992) Improving communication with cancer patients. Oncology 6: 83–89 1390017

[bib24] Smith CK, Polis E, Hadac RR (1981) Characteristics of the initial medical interview associated with patients satisfaction and understanding. J Fam Pract 12: 283–288 7462936

[bib25] Spiro H (1992) What is empathy and can it be taught? Ann Intern Med 116: 843–846 1482433 10.7326/0003-4819-116-10-843

[bib26] Stiles WD, Putnam SM, Wolf MH, Sherman JA (1979) Interaction exchange structure and patient satisfaction with medical interviews. Med Care 17: 667–679 449436 10.1097/00005650-197906000-00010

[bib27] Wiggers J, ÓDonovan K, Redman S, Sanson-Fisher R (1990) Cancer patient satisfaction with care. Cancer 66: 610–616 2364373 10.1002/1097-0142(19900801)66:3<610::aid-cncr2820660335>3.0.co;2-t

